# Lesion-Specific Immune Response in Granulomas of Patients with Pulmonary Tuberculosis: A Pilot Study

**DOI:** 10.1371/journal.pone.0132249

**Published:** 2015-07-02

**Authors:** Selvakumar Subbian, Liana Tsenova, Mi-Jeong Kim, Helen C. Wainwright, Annalie Visser, Nirmalya Bandyopadhyay, Joel S. Bader, Petros C. Karakousis, Gabriele B. Murrmann, Linda-Gail Bekker, David G. Russell, Gilla Kaplan

**Affiliations:** 1 Laboratory of Mycobacterial Immunity and Pathogenesis, Public Health Research Institute (PHRI), Rutgers Biomedical and Health Sciences, Rutgers, The State University of New Jersey, Newark, New Jersey, United States of America; 2 Department of Biological Sciences, NYC College of Technology, Brooklyn, New York, United States of America; 3 Department of Immunobiology, Joslin Diabetes Center, Harvard Medical School, Boston, Massachusetts, United States of America; 4 Division of Anatomical Pathology, Faculty of Health Sciences, University of Cape Town, Cape Town, South Africa; 5 Department of Biomedical Engineering, High-Throughput Biology Center and Institute of Computational Medicine, Johns Hopkins University, Baltimore, Maryland, United States of America; 6 Center for Tuberculosis Research, Department of Medicine, Johns Hopkins University School of Medicine and Department of International Health, Johns Hopkins Bloomberg School of Public Health, Baltimore, Maryland, United States of America; 7 Department of General and Thoracic Surgery, Medisch Centrum Leeuwarden, Leeuwarden, The Netherlands; 8 The Desmond Tutu HIV Centre, Institute of Infectious Disease and Molecular Medicine and Department of Medicine, University of Cape Town, Cape Town, South Africa; 9 Department of Microbiology and Immunology, College of Veterinary Medicine, Cornell University, Ithaca, New York, United States of America; 10 Bill and Melinda Gates Foundation, Seattle, Washington, United States of America; Institut Pasteur, FRANCE

## Abstract

The formation and maintenance of granulomas is central to the host response to *Mycobacterium tuberculosis* (Mtb) infection. It is widely accepted that the lungs of patients with tuberculosis (TB) usually contain multiple infection foci, and that the granulomas evolve and differentiate independently, resulting in considerable heterogeneity. Although gene expression profiles of human blood cells have been proposed as biomarkers of Mtb infection and/or active disease, the immune profiles of discrete lesion types has not been studied extensively. Using histology, immunopathology and genome-wide transcriptome analysis, we explored the immunological profile of human lung TB granulomas. We show that although the different granulomas share core similarities in their immunological/inflammatory characteristics, they also exhibit significant divergence. Despite similar numbers of CD68^+^ macrophages in the different lesions, the extent of immune reactivity, as determined by the density of CD3^+^ T cells in the macrophage rich areas, and the extent of fibrosis, shows considerable variation. Both quantitative and qualitative differences among significantly differentially expressed genes (SDEG) were noted in each of the lesion types studied. Further, network/pathway analysis of SDEG revealed differential regulation of inflammatory response, immune cell trafficking, and cell mediated immune response in the different lesions. Our data highlight the formidable challenges facing ongoing efforts to identify peripheral blood biomarkers due to the diversity of lesion types and complexity of local immune responses in the lung.

## Introduction

Tuberculosis (TB), caused by infection with *Mycobacterium tuberculosis* (Mtb) was responsible for about 1.5 million deaths and 9 million new cases worldwide in 2013 http://www.who.int/mediacentre/factsheets/fs104/en/. Following inhalation of virulent Mtb-containing aerosol droplets into the alveoli of the lung, the bacteria are phagocytosed by resident macrophages that produce and secrete cytokines, chemokines and other inflammatory mediators that recruit additional leukocytes from the circulation to the site of infection [1],[2]. Recruitment of leukocytes around infected macrophages results in the formation of granulomas, which are highly cellular structures that contribute to limiting the spread of Mtb [3,4]. The course of evolution of the granulomas is determined locally by the balance between host- and pathogen-derived factors. In most cases, at the center of the granuloma, heavily infected macrophages that are unable to control bacillary growth undergo necrosis, releasing Mtb as well as host cell contents into the extracellular milieu. These bacilli can be phagocytosed by newly arriving macrophages, resulting in further recruitment and activation of immune cells, and the formation of larger and more differentiated granulomas [5,6]. The necrotic center in granulomas may undergo caseation and liquefaction, ultimately leading to cavity formation [7,8]. Cavitation facilitates dissemination of the infecting bacilli from the granuloma via the airways to the external environment [9,10]. Consequently, granulomas play a crucial role in the control of Mtb infection and establishment of latency (LTBI) as well as in the spread of the disease (active TB). Importantly, granulomas in Mtb-infected human lungs mature and evolve independently of each other, determined primarily by local immunity and bacterial growth [11–14]. We have previously shown the presence of structurally diverse granulomatous lesions within the same lungs of TB patients [7] [9].

Although gene expression profiles from the host blood and bronchoalveolar lavage (BAL) cells have been used as biomarkers to differentiate, classify and characterize the spectrum of active TB versus LTBI, only a few studies have explored the immune environment within lung granulomas [7,12,15–20]. In these studies, the presence of distinct subsets of immune cells, including macrophages expressing nitric oxide synthase and arginase, DEC-205^+^ foamy macrophages, CD5^+^/CD19^+^ effector lymphocytes and Treg cells, as well as differential vascularization patterns, revealed through CD31 and Ki-67 staining, have been reported to delineate and define different microenvironments in the granulomas of pulmonary TB patients [12,16,17,21]. Recently, using molecular and biochemical methods, we reported enhanced host lipid metabolism and induction of macrophage endoplasmic reticulum stress, which correlated with caseation of granulomas in the lungs of human TB patients [7,22]. In the present study, using histopathologic analysis, immune cell enumeration and genome-wide transcription profiling, we studied the local immune environment associated with different types of granulomas in the lungs of TB patients, including paucibacillary fibrotic nodules and cavities with either few or numerous acid fast bacilli (AFB).

## Materials and Methods

### Human TB subjects and ethics statement

Informed, written consent was obtained from six patients recruited into this study. The patients were admitted at Groote Schuur Hospital, Cape Town, South Africa, between January 2000 and December 2001 for lobectomy because of poor response to anti-TB drug treatment or due to post-TB complications as previously described [9]. All patients in this study were HIV-negative. Standard preoperative procedures, including diagnostic Mtb culture from sputum and chest radiography, were performed for all patients. The procedures to recruit patients, consent letters to collect, process and analyze tissue were approved by the Health Sciences Ethics Committee, University of Cape Town, South Africa, and by the institutional review boards at the Public Health Research Institute, UMDNJ, Newark, NJ and Cornell University, Ithaca, NY.

### Types of lung TB granulomas studied

Among the six patients studied, two had multidrug resistant (MDR) TB. Three patients were sputum culture-positive, despite 18–24 months of anti-TB therapy as previously described [9]. The other three patients had previously been treated for pulmonary TB and were sputum culture-negative. However, these patients showed symptoms of possible relapse, including hemoptysis. Patients were retreated empirically for TB for 7–15 months before lung surgery. Each of the resected lungs had multiple and diverse types of granulomatous lesions, ranging from small cellular granulomas to large cavities.

### Lung tissue processing

The surgically resected human TB lung specimens were transferred to a biological safety level-3 (BSL3) facility for gross pathological examination and dissection of macroscopic granulomatous lesions as previously described [9]. Representative samples of the different types of granulomas were collected for study. Portions of the lungs were fixed with 10% formalin and embedded in paraffin, sectioned at 2 μm thickness and stained with hematoxylin and eosin (H&E) or with carbolfuchsin to visualize AFB. The bacillary load was scored as none (no countable bacilli), scanty (individual bacilli found in some, but not in all fields of vision), moderate (1 to 10 bacilli in each field of a lesion), or numerous (more than 10 bacilli, mostly clumps, found in each field of a lesion).

### Immunohistochemical (IHC) staining

For IHC staining, a total of 18 sections of non-necrotic cellular lesions from 4 granulomas, 13 sections of necrotic lesions from 7 granulomas, 13 sections from 3 fibrotic granulomas and 16 sections of the cavity wall from 9 granulomas, were analyzed for immune cell composition. The paraffin-embedded sections were placed on Superfrost/Plus glass slides (Fisher Scientific, Pittsburgh, PA), deparaffinized, rehydrated and subjected to antigen retrieval by boiling in 0.1 M citrate buffer, pH 7.0 (for CD3, CD8 and CD68), or in 0.1 M EDTA buffer, pH 7.0 (for CD4). Monoclonal antibodies for human anti-CD3 (Ventana, Tucson, AZ), anti-CD4 (Nova Castra, New Castle upon Tyne, United Kingdom), anti-CD8 and anti-CD68 (Dako, Carpinteria, CA) were used at 1:100, 1:20, 1:20 and 1:500 dilutions, respectively, to determine the immune cell distribution. The staining was performed in an automated immunostainer (Ventana, Tucson, AZ) using an immunoperoxidase-diaminobenzidene (DAB) kit (Ventana, Tucson, AZ) as reported earlier [23]. Though both macrophages and MNGs can stain positive for CD68, the histologic sections were analyzed manually under microscope to discriminate MNGs with giant and multi-nucleated appearance from macrophages.

### Laser capture micro-dissection (LCM)

The LCM was performed as described before [7]. Briefly, 500 mg of lung segments were snap frozen, embedded in Tissue-Tek O.C.T Compound (Sakura Finetek USA, Inc., Torrance, CA) and cut into 10 μm-thick sections on a Cryocut 1800 cryostat (Leica Microsystems Inc, Buffalo Grove, IL). The sections were mounted onto PET-membrane slides (Leica Microsystems Inc, Buffalo Grove, IL) for LCM, and Superfrost/Plus glass slides (Erie Scientific, Portsmouth, NH) for histological analysis. The histology slides were fixed in 4% paraformaldehyde and stained with hematoxylin/eosin, and paired with the slides prepared for LCM to select the cellular areas to be studied. For LCM the sections were fixed in ethanol gradient (70, 75, 96 and 100%) containing 0.5% sodium azide, air dried briefly and areas of interest were dissected by using Leica AS LMD system (Leica Microsystems Inc, Buffalo Grove, IL).

### RNA isolation for microarray

The RNA from lung granulomas for transcriptional data was from our previous study [7]. Briefly, for the microarray gene expression studies, LCM material, obtained from 4 granulomas was used for total RNA isolation. The lesions selected for transcriptional analysis were: 2 small caseous/necrotic nodules with fibrotic encapsulation and scanty AFB and 2 open cavitary lesions with variable numbers of AFB. The uninvolved lung parenchyma from 3 sputum-AFB-negative, previously infected and fully treated TB patients, who underwent resections due to post-TB complications, was collected and used as controls. We performed three independent microarrays on one of the 2 LCM-derived fibrotic lesions, which confirmed the reliability of the technique. Another fibrotic lesion and 2 cavitary granulomas (one with high AFB and the other with scanty AFB) were also analyzed by microarray.

The microarray experiments using total RNA from human lung granuloma sections were performed as described earlier [7]. Briefly, total RNA isolated from tissue sections obtained from LCM was treated with DNase and purified RNA was amplified, labeled and hybridized onto GeneChip Human X3P Array (Affymetrix, Santa Clara, CA). Arrays were washed and scanned in a GeneArray 300 scanner (Affymetrix, Santa Clara, CA). Three microarray experiments were performed for each of the LCM-derived human TB lung sections. Total RNA generated from three independent LCMs was processed separately for the array experiments. The array data was validated by qRT-PCR and published in a previous manuscript [7]. Microarray data was submitted to Gene Expression Omnibus (GEO accession number GSE20050) [7].

### Microarray data analysis

The CEL files obtained from microarrays was processed as reported previously [7,24]. Briefly, probe level analysis of CEL files was performed using R and Bioconductor and a list of gene expressions in various granulomas was obtained. Raw intensities of perfectly matching (PM) probes expressed in each array were subjected to local background correction using Microarray Suite Version 5.0 software (MAS5; Affymetrix, Santa Clara, CA). The values from each sample (in triplicate) were log_2_-transformed and median-centered. A family-wise *p*-value of ≤ 0.05 was applied to select the list of significantly differentially expressed genes (SDEG) between lung granulomatous lesion and uninvolved (control) parenchyma from raw CHP files using Partek Genomics Suite Version 6.7 software (Partek, St. Louis, MO). The expression levels in various granulomas were normalized with the corresponding gene expression in the uninvolved parenchyma. Gene ontology analysis and intensity plots of SDEG expressed exclusively or commonly between different types of lung granulomas were generated using Partek Genomics Suite Version 6.7 software (Partek, St. Louis, MO) as described previously [25]. The SDEG were further analyzed to determine pathway/network significantly affected by SDEG using Ingenuity Pathway Analysis (IPA) software (Ingenuity Systems, Redwood City, CA).

### Statistical analysis

One-way ANOVA with equal variance was applied to background-corrected microarray data from granulomatous lesions and control lung tissue and a false discovery rate of 5% (*q* = 0.05) was set as cutoff to select SDEG in Partek Genomics Suite Version 6.7 software. The statistical significance for pathway/network analysis was calculated using right-tailed Fisher Exact Test and a *p*-value ≤0.05 was considered significant for a pathway.

## Results and Discussion

### Global gene expression pattern of human TB lung granulomas

To determine the host immune environment within the lung granulomas of TB patients, 4 granulomas were selected during macroscopic dissection and submitted for gene expression analysis and histological analysis (see methods). The transcriptome of these independently selected lesions (n = 4) were pooled and analyzed, relative to the pooled transcriptome from un-involved control tissue segments (n = 3). Our results show that in the TB lung granulomas, of the 11,651 SDEG, a total of 4,462 (38%) genes were up-regulated, while 7,189 (62%) genes were down-regulated, relative to the uninvolved lung tissue. A z-score based gene ontology (GO) analysis of these SDEG revealed activation (z > +2) of biological functions associated with inflammation, cell death, immune cell movement, cell mediated immunity, cell communication and signaling in the granulomatous lesions (Table 1). Transcripts linked to production of nitric oxide and reactive oxygen species in macrophages were among the top 10 activated canonical pathways (S1 Table). Other pathways of interest included B cell receptor signaling, PKCθ signaling in T lymphocytes and integrin signaling. Consistently, genes involved in tissue damage and remodeling, such as *MMP1*, *MMP9* as well as inflammatory chemokines, including *CXCR4*, *CCL3* (MIP-1alpha) and *CXCL8* (*IL8*) were among the top 10 highly up-regulated SDEG, while several transcriptional regulators (*FOXC1*, *NFLX*, *ERG*, *ATN1* and *NEUROG1*) and enzymes (*PIN1*, *AKT3*, *ERBB3* and *EGFR*) were among the most highly down-regulated genes (S2 Table). Taken together, the global gene expression analyses show significant up regulation of inflammation, cellular immunity and tissue damage networks in the lung granulomas of active TB patients. These observations are consistent with previous reports showing elevated expression of CXCR-4, CCL-3, CXCL-8, MMP-1 and -9 (matrix metalloproteinases) in the sputum, blood, lymph node, bronchoalveolar lavage (BAL) fluid and lungs of chronic, progressive pulmonary TB patients [19,26–31]. Importantly, mRNA for the most highly expressed inflammatory proteins included MMPs, CCL3 and CXCL8, which have previously been shown to be capable of driving the maturation/cavitation of lung TB granulomas and disease progression [17,28,32,33]. Though the level of response to chemotherapy among TB patients was not evaluated in this study, factors such as the nature of infecting Mtb strain, extent of disease and the mode of TB onset (i.e, reinfection or reactivation) in these patients were inherent to pulmonary TB in humans and are very difficult, if not impossible, to ascertain.

**Table 1:** Gene ontology (GO) analysis of SDEG in TB lung granulomas.

No.	Functional categories	z-score	p-Value	# of Molecules
***Upregulated/Activated functions***				
1	Infectious Disease	7.002	8.88E-16	345
2	Cell Death and Survival	6.015	1.38E-15	632
3	Immune Cell Trafficking	5.661	2.33E-14	428
4	Inflammatory Response	5.422	6.62E-09	258
5	Hematological System Development and Function	5.313	1.20E-07	172
6	Organismal Injury and Abnormalities	5.27	1.93E-11	136
7	Cellular Function and Maintenance	5.1	2.71E-14	207
8	Cell-mediated Immune Response	4.633	1.10E-15	255
9	Cellular Compromise	4.594	4.96E-08	82
10	Cell-To-Cell Signaling and Interaction	4.425	4.09E-15	349
***Downregulated / Inhibited functions***				
11	Gastrointestinal Disease	-2.153	9.10E-22	1854
12	Connective Tissue Disorders	-2.32	1.10E-07	40
13	Reproductive System Disease	-2.346	2.27E-27	1525
14	Digestive System Development and Function	-2.387	4.87E-13	288
15	Cell Morphology	-2.628	7.13E-15	214
16	Hepatic System Development and Function	-3.502	8.83E-09	129
17	Cancer	-3.663	9.22E-09	146
18	Organ Development	-3.69	8.06E-09	132
19	Developmental Disorder	-4.692	6.27E-09	82
20	Organismal Survival	-7.756	2.80E-28	1088

### Histopathological analysis of selected human lung TB granulomas

**Fig 1:**
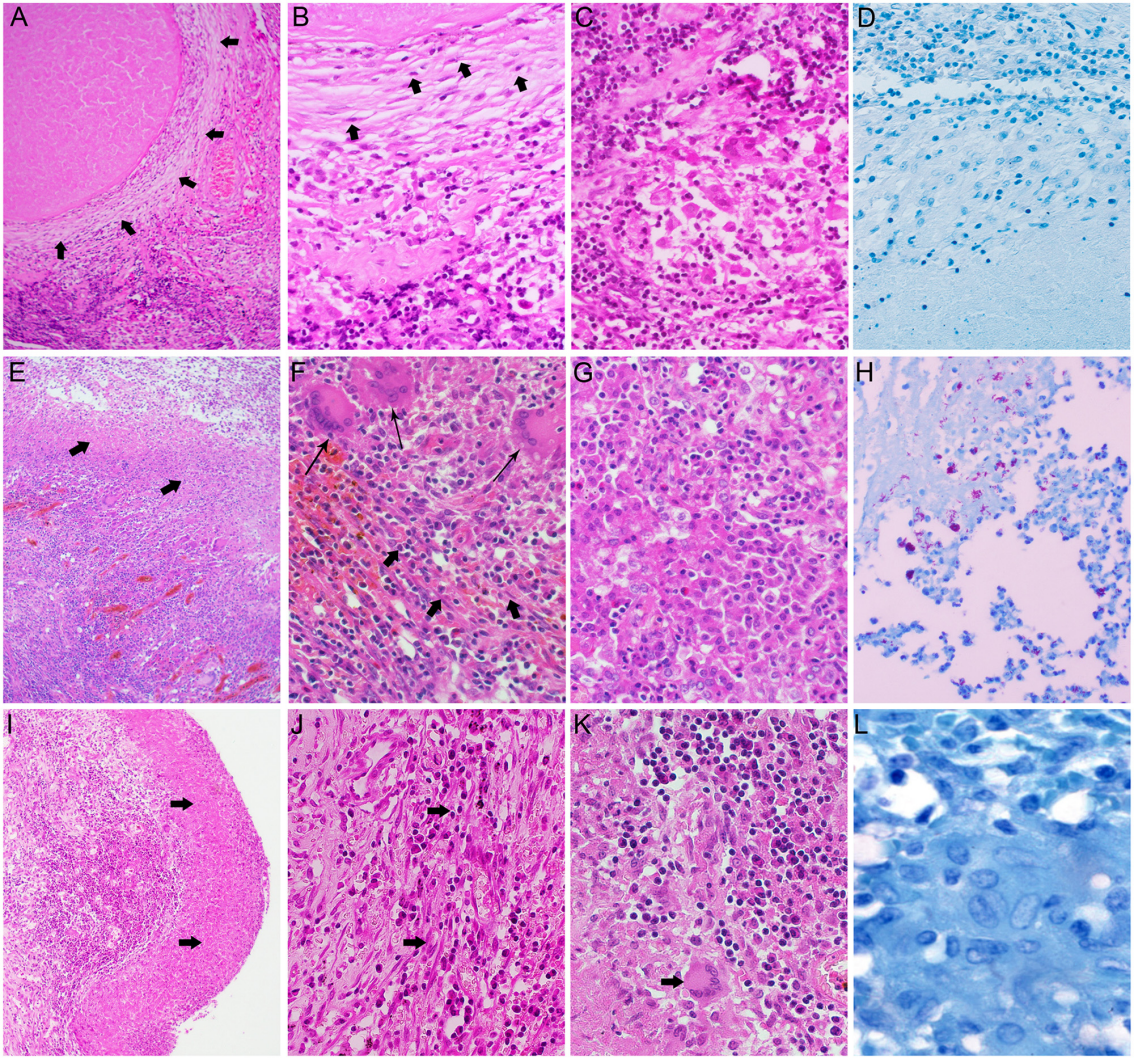
Histopathology of granulomas in the lungs of TB patients. H&E and AFB stained lung sections from TB patients showing various types of granulomatous lesions. (A-D). H&E stained section of a closed fibrotic caseous nodule showing a necrotic center with a thickened fibrous wall (arrows in A), composed of fibroblasts (arrows in B) and scattered leukocytes. (C). The fibrotic area was surrounded by a rim of loosely accumulated macrophages, interspersed with T cells and few MNG. (D). No AFB was detected in these lesions. (E-H). H&E stained sections of an open cavitary lesion showing necrotic cellular debris (arrows in E) surrounded by a layer of activated epithelioid macrophages, MNG cells (thin arrows in F), and numerous scattered lymphocytes (F and G). (H). Abundant AFB were detected at the luminal surface of the cavity. (I-L). H&E stained section of a cavitary lesion showing necrotic debris (arrows in I) surrounded by layer (arrows in J) composed of macrophages, MNG (arrow in K) and lymphocytes (J and K). (L). Few if any AFB were detected at the luminal surface of the cavity. Magnification: A, E, I 4x; B-D, F-H, J-K, 40x; L, 100x.

To define the cellular architecture of the granulomas selected for gene expression analysis, we performed a histological analysis of H&E-stained lung sections. All lesions contained a zone of caseous necrosis, surrounded by a highly cellular area (Fig 1A, 1E and 1I). However, the cellular organization differed among the different lesions. Two of the lesions selected were fibrotic nodules with similar architecture (Fig 1A–1D) and two were segments of the wall of open cavitary lesions (Fig 1E–1L). The closed fibrotic nodules were characterized by a necrotic center with a clear fibrous rim containing multiple fibroblasts and scattered mononuclear leukocytes (Fig 1A & 1B). The fibrotic area was surrounded by loosely accumulated macrophages, interspersed with T cells and few multinucleated giant cells (MNG) (Fig 1C). No liquefaction or cavitation was noted. As reported previously, no AFB was seen in these nodules (Fig 1D and [9]). In contrast, the two cavitary lesions contained a necrotic rim, surrounded by a layer of activated epithelioid macrophages, MNG, and numerous scattered lymphocytes (Fig 1E–1G and 1I–1K). In some areas of the cellular zone, loose fibrotic deposition and layering of the cells was noted (Fig 1G and 1K). Large numbers of AFB were detected at the luminal surface of the cavity of one of the selected lesions (Fig 1H). In contrast, in spite of a similar immune cell distribution in the second cavitary lesion (Fig 1I–1K), very few or no detectable AFB (Fig 1L) was found. The uninvolved lung parenchyma contained small numbers of alveolar macrophages and very few, if any, T cells (S1 Fig).

Studies addressing the independent evolution of different granulomas from pulmonary TB patients are limited by the paucity of freshly-resected lung tissues [9]. However, the heterogeneity of lung lesions observed in chronic TB patients has also been reported in the rabbit and nonhuman primate model of active pulmonary TB, providing an alternative source of tissue for in-depth analyses of the various microenvironments that can be found in the lungs during chronic TB disease [34–36]. Moreover, molecular techniques, such as in situ hybridization and RNA-seq should now facilitate more extensive interrogation of formalin-fixed tissues processed for histopathologic analysis, providing an additional source of material to be used for future analysis of the complexity of granuloma maturation and differentiation in the human lung.

### Immune cell distribution in the different types of TB granulomas

To extend the histopathological examination and to determine the level of heterogeneity in cellular phenotype in different pulmonary TB granulomas, we performed immunohistochemical staining (IHC) on multiple lesions (see methods). The distribution of mononuclear leukocytes in different types of granulomas was quantified using a microscopy-based morphometric analysis of the sections stained for cell surface markers of macrophages (CD68^+^), MNG (CD68^+^), and T lymphocytes (CD3^+^, CD4^+^ or CD8^+^). Our analysis revealed similar numbers of macrophages and MNGs in all the types of granulomas examined (Table 2). There was no association between the presence/abundance of AFB and the number of these cells. However, there was a clear heterogeneous distribution of T lymphocytes (CD3^+^) in the different granulomas. Calcified fibrotic nodules, indicative of resorbing non-active lesions, contained fewer CD3^+^ T cells. In contrast, small closed necrotic and non-necrotic cellular granulomas as well as open granulomas with central cavities contained higher numbers of CD3^+^ and CD4^+^ T cells, respectively. The uninvolved lung parenchyma contained very few CD3^+^ and CD68^+^ mononuclear cells and no CD68^+^ MNGs (Table 2). As previously reported, numerous CD68^+^ macrophages and polymorphonuclear leukocytes (PMN) were present at the cavity surface (not shown) [9]. However, no T cells were found at the luminal surface of cavities and in the necrotic zones, thereby preventing direct T cell-macrophage interactions at those sites. The differential leukocyte distribution suggests that some granulomatous structures of the TB lung are immunologically reactive with more cell recruitment and/or replication than others and represent distinct microenvironments.

**Table 2:** Distribution of immune cells in different type of TB lung granulomas*.

Lesion Type	CD68^+^ **	CD68^+^ MNG***	CD3^+^	CD4^+^	CD8^+^
Cavity (open granuloma	29.7 (4.48)	1.45 (0.05)	109.45 (14.53)	39.5 (6.46)	27.5 (7.12)
Necrotic (non-cavitary)	25.6 (3.18)	1.5 (0.15)	57.9 (10.27)	21.4 (3.58)	25.9 (4.53)
Non-Necrotic (cellular)	27.5 (2.68)	3 (0.72)	74 (9.17)	31.5 (6.07)	19 (3.44)
Fibrotic nodule	25 (2.5)	1.2 (0.12)	14 (1.4)	n/d	n/d
Uninvolved parenchyma	6 (0.6)	n/f	6 (0.6)	n/f	n/f

Average number of cells +/− SEM per x100 microscopic field; 3–7 fields per tissue sample;

* cell counts are independent of bacillary load for cavitary lesion;

**all CD68^+^ cells;

*** multinucleated giant cells;

n/d—not determined; n/f-not found

Consistent with our immune phenotyping findings, the heterogeneity in the presence of CD4^+^ and CD8^+^ T lymphocytes and their effecter functions during Mtb infection has been reported previously [1,3,12,37–40]. In addition, a significant increase in the percent of activated leukocytes, including CD3^+^/HLA-DR^+^, CD4^+^ and CD8^+^ T cells has been reported in the BAL fluid and in the cavitary lung lesions, compared to non-cavitary granulomas, in patients with pulmonary TB [12,16,21,41,42]. Macrophages play crucial role in the outcome following Mtb infection in humans [43]. Significant elevation in the distribution of CD68^+^ macrophages was observed in the lymph nodes of TB patients [44]. These CD68^+^ macrophages that also contain Mtb antigens were the major source of elevated iNOS (inducible nitric oxide synthase) production in the granulomas [45].

### Cavitary granulomas and fibrotic nodules of human lungs display distinct gene expression profiles

**Fig 2:**
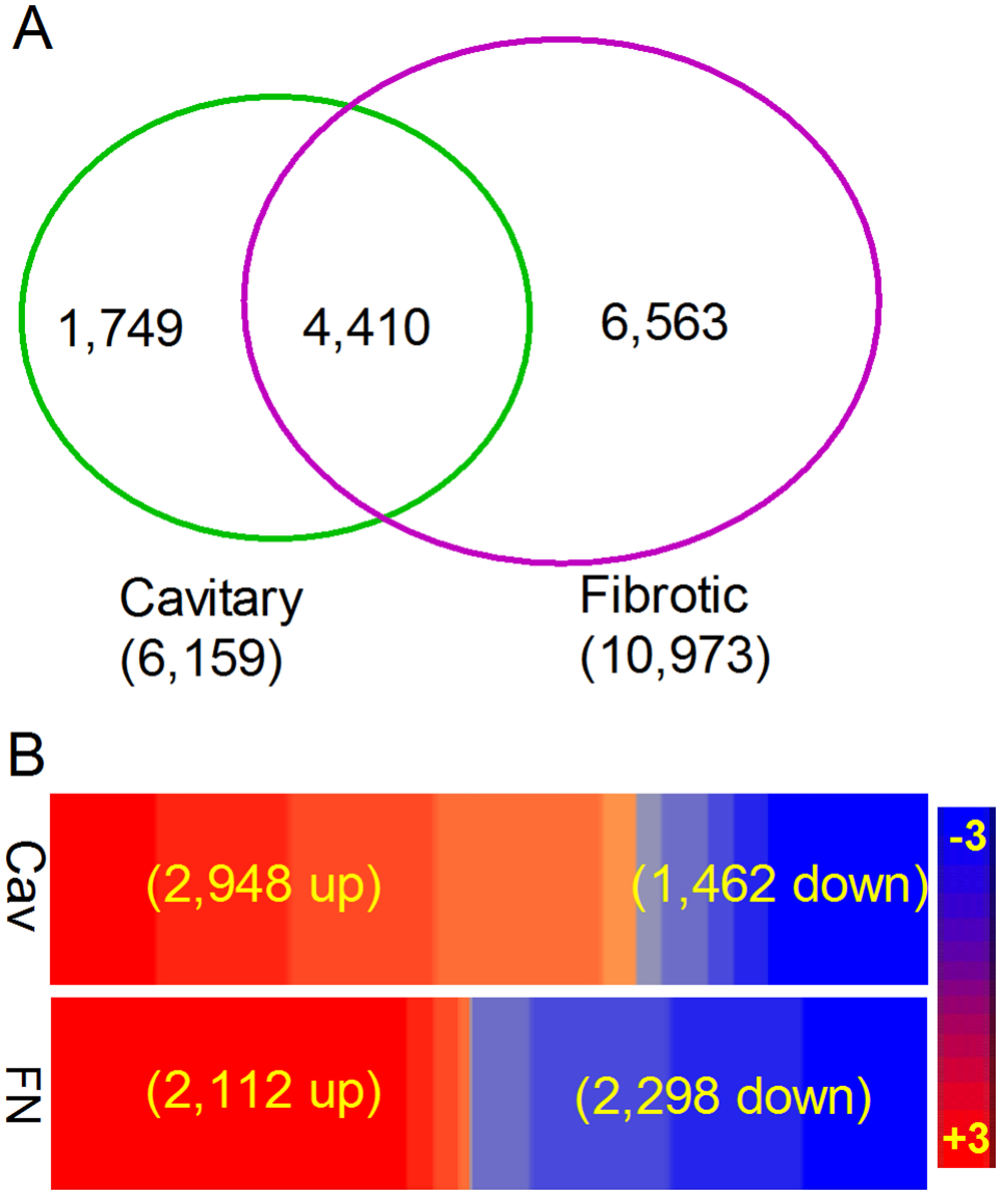
Overview of host gene expression profile in the cavitary and fibrotic granulomas of human TB lungs. (A). Venn diagram showing the number of unique or shared SDEG between the cavitary granulomas (green circle) and fibrotic nodules (purple circle). Numbers in parenthesis shows total number of SDEG. (B). Intensity plot showing the expression pattern of shared SDEG between cavitary (Cav) and fibrotic (FN) granulomas. Up-regulated SDEG are in red and down-regulated are in blue. Expression patterns are sorted in descending order (left to right). Numbers in parenthesis shows the up- and down-regulated SDEG in each lesion type. The scale bar ranges from +3 (red) to -3 (blue).

Our histological analysis and enumeration of immune cells suggested that a pooled transcriptome analysis of different granulomas may underestimate subtle differences in gene expression specific to each of the lesion types shown in Fig 1 and Table 2. Therefore, to determine the association between the molecular correlates of immune response and the extent of immune cell distribution/activation in the different types of lung granulomas, we compared the gene expression profiles between pooled cavitary granulomas (n = 2) and fibrotic nodules (n = 2), relative to uninvolved control tissue (n = 3). As shown in Fig 2, there was a higher number of SDEG expressed in the fibrotic nodules, compared to the cavitary granulomas (10,973 versus 6,159). Among the unique SDEG, about a four-fold higher number was noted in the fibrotic nodules, compared to cavitary granulomas (6,563 versus 1,749) (Fig 2A). Expression of 4,410 SDEG was commonly regulated between these two lesion types (Fig 2A and 2B). Of these, 2,948 (~67%) and 2,112 (~48%) SDEG were up-regulated in the fibrotic nodules and cavitary granulomas, respectively (Fig 2B and S3 Table). Interestingly, of the common SDEG, a total of 1,185 genes were up-regulated by more than five-fold in the cavitary granulomas, compared to only 17 genes in the fibrotic nodules (S3 Table). Thus, a clearly distinct gene expression pattern was observed for cavitary lesion versus fibrotic granulomas.

To identify the biological functions affected by the SDEG in cavitary granulomas and fibrotic nodules, we performed gene ontology (GO) analysis. As shown in Table 3, twelve of the top 25 most significantly affected biological functions were directly associated with the host immune response, including cytokine and chemokine signaling and their downstream processes such as antigen presentation and lysosome function. However, the spectrum of significantly-involved (*p*<0.05) biological functions was different between the cavitary granulomas and fibrotic nodules. While chemokine signaling, NOD-like receptor signaling, cytokine-cytokine receptor interaction, and chemokine-chemokine receptor signaling networks were the most affected in cavitary granulomas, MHC-class I-mediated antigen processing and presentation and cell adhesion molecule networks/pathways were affected to a greater extent in the fibrotic nodules (Table 3). As reported earlier, metabolism of lipids and lipoproteins were among the top 5 most significantly affected networks in both types of granulomatous lesions [7].

**Table 3:** Pathway/Network analysis of SDEG in TB lung granulomas with different bacillary load.

	p-value			
Pathway/Network	Gene Count	All	Cavity (AFB-H) *	Cavity (AFB-S) **
Reactome_Cytokine signaling in immune system	260	1.37E-114	4.50E-116	1.96E-103
Reactome_Interferon signaling	150	5.81E-82	6.37E-81	2.67E-79
KEGG_Lysosome	119	1.23E-81	5.61E-70	1.91E-115
Reactome_Class_I_MHC mediated antigen processing and presentation	234	2.02E-81	4.43E-76	1.48E-67
Reactome _Metabolism of lipids and lipoproteins	459	6.77E-76	8.01E-71	4.96E-73
KEGG_Graft versus host disease	36	1.05E-67	1.52E-46	7.65E-62
KEGG_Type_I_diabetes mellitus	42	1.94E-65	3.59E-45	9.06E-64
Reactome _Hemostasis	433	2.20E-65	2.57E-61	4.41E-73
Reactome E_Interferon gamma signaling	57	1.34E-64	1.89E-57	6.66E-62
Reactome E_Innate immune system	246	1.19E-63	1.25E-57	1.81E-62
Reactome _Cell cycle	371	9.13E-63	2.84E-54	5.21E-50
KEGG_Antigen processing and presentation	79	2.25E-61	2.64E-46	2.19E-74
KEGG_Leishmania infection	69	1.22E-58	2.01E-39	1.14E-59
KEGG_NOD_like receptor signaling pathway	62	6.07E-58	2.60E-65	3.20E-47
KEGG_Cytokine-cytokine receptor interaction	251	1.19E-56	9.81E-55	1.11E-33
KEGG_Aallograft rejection	36	4.27E-56	2.80E-37	4.24E-56
KEGG_Pathways in cancer	323	1.19E-55	5.57E-53	9.10E-44
Reactome _Developmental biology	375	4.21E-55	3.66E-50	1.84E-51
Reactome _HIV_Infection	189	5.08E-55	2.55E-47	5.48E-55
KEGG_Cell adhesion molecules	131	1.39E-54	3.36E-41	8.38E-50
Reactome _Mitotic cell cycle	303	4.96E-54	2.51E-44	5.34E-41
KEGG_Chemokine signaling pathway	178	6.23E-53	2.11E-58	7.06E-39
Reactome _Antigen processing_ubiquitination_proteasome degradation	197	3.20E-51	1.06E-46	3.31E-38
Reactome _Chemokine receptors_bind_chemokines	53	4.57E-51	5.28E-47	1.81E-22

* cavitary lesion with high bacillary load;

**cavitary lesion with scanty bacillary load.

### Differential regulation of selected network/pathway genes in the cavitary granuloma and fibrotic nodules of human pulmonary TB

**Fig 3:**
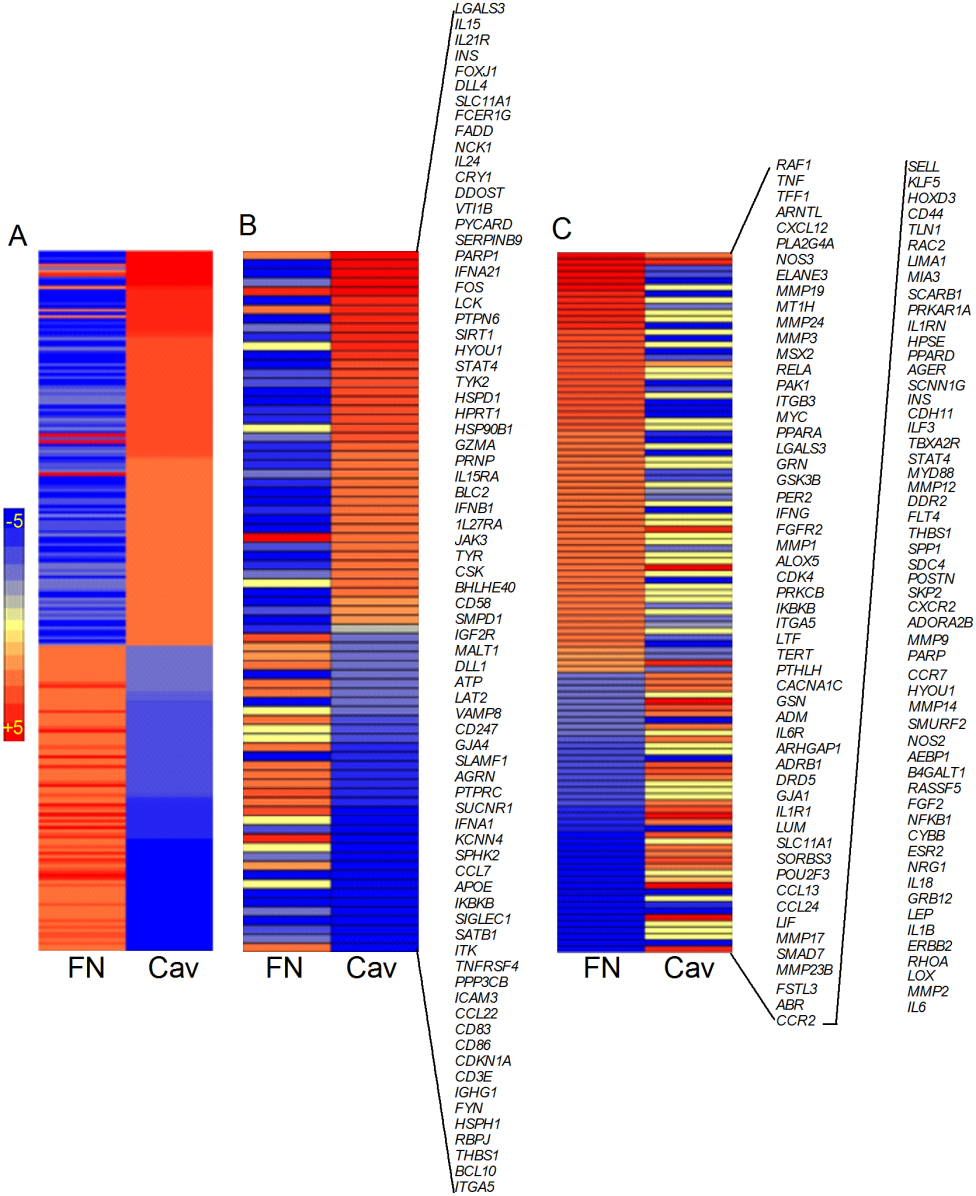
Network analysis of SDEG in the lung cavitary (Cav) and fibrotic (FN) granulomas. (A). Intensity plot showing the expression pattern of up-regulated (red) and down-regulated (blue) SDEG in the immune cell movement network. Expression pattern in cavitary lesions is sorted in descending order (top to bottom). (B). Intensity plot showing the expression pattern of SDEG and their gene symbols in the STAT1-mediated T cell activation network. Expression pattern in cavitary lesions is sorted in descending order (top to bottom). (C). Intensity plot showing the expression pattern of SDEG and their gene symbols in the fibrosis and wound healing network. Expression pattern in fibrotic lesions is sorted in descending order (top to bottom). For A, B and C, red color indicates up-regulated and blue color represent down-regulated SDEG and the scale bar ranges from +5 (red) to -5 (blue).

To start to build linkages between SDEG and structurally-diverse granulomas, we explored the network/pathway associated with selected cellular functions. We selected three networks/pathways based on the GO analysis of SDEG and the immune cell distribution revealed by our histological analyses of the cavitary granulomas and fibrotic nodules. The selected gene networks included those associated with immune cell movement, STAT1-mediated T cell activation and, fibrosis and wound healing (Fig 3A–3C and S4 Table). In addition, we analyzed the gene expression pattern of vitamin D receptor (VDR) signaling and IL-17 interaction networks due to their crucial role in the immune response to Mtb infection [46] [47] (S2 and S3 Figs and S5 Table).

#### Immune cell movement network

Among the SDEG commonly expressed in the cavitary granulomas and fibrotic nodules, a subset of 280 genes were associated with immune cell movement, a host cell response to Mtb infection and tissue injury. As shown in the intensity map, more SDEG was up-regulated by more than 2-fold in the cavitary granulomas, relative to the fibrotic nodules (n = 123 versus 104 genes). In addition, more than 50% of SDEG in this network were expressed in opposite directions between cavitary granulomas and fibrotic nodules (Fig 3A and S4 Table).

#### STAT1-mediated T cell activation network

Of the 76 SDEG associated with the STAT1-mediated T cell activation network, expression of 37 and 17 genes was up-regulated by greater than 2-fold in the cavitary granulomas and fibrotic nodules, respectively. In addition, 34 and 46 genes were down-regulated by greater than 2-fold in the cavitary granulomas and fibrotic nodules, respectively (Fig 3B and S4 Table). Notably, compared to fibrotic nodules, expression of genes associated with T cell activation, including transcriptional regulators, *STAT1*, *STAT4*, *JAK3*, *CSK* and *FADD*, and cytokines *IL15*, *IL15RA*, *IL21R*, *IL27RA*, *FADD*, *IFNA21*, *IFNB1* and *GZMA* was significantly up-regulated only in the cavitary granulomas. In addition, 10 of the 76 SDEG in the T cell activation network, including *IL24*, *FOS*, *TYR*, *AGRN*, *SUCNR1*, *IFNA1*, *TNFRSF4*, *CCL22* and *CD3E* were either not expressed or not significantly differentially expressed in the fibrotic nodules, relative to uninvolved lung parenchyma. Consistent with our findings, a significant increase in the expression of *GZMA* (granzyme A) and in the number of GZMA-expressing CD3^+^ cells has been reported in lung granulomas, compared to un-involved parenchyma of cavitary TB patients [45]. In humans, GZMA, along with other inflammatory molecules, such as interleukins and interferons, have been shown to activate tissue damage and pathology during HIV-infection, leishmaniasis and TB [48,49].

#### Fibrosis and wound healing network

There were 110 SDEG associated with the tissue fibrosis and wound healing network (Fig 3C and S4 Table). Of these, expression of 60 genes was up-regulated and 43 down-regulated, by more than 2-fold in the fibrotic nodules. The number of SDEG up-regulated and down-regulated was reduced to 27 and 36, respectively, in the cavitary granulomas. In addition, the expression level of 41 genes was significant only in the fibrotic nodules compared to the cavitary granulomas. In conclusion, an association was noted between the type of TB lesion and changes in the expression pattern of selected network/pathway genes. The expression profile of network genes associated with immune cell movement, T cell activation, and fibrosis and wound healing were consistent with and supported by the histological analysis of the respective lung TB lesions. Similar to our observation, recent studies on TB patients have shown significant up-regulation of several host genes, including *IFNGR2*, *IRF1*, *IFIT3*, *IFITM1*, *SOCS1*, *TAP1*, *SPP1* and *STAT1* at the local (infected lung tissue) and systemic (blood) level only during active pulmonary TB and not in LTBI or in healthy contacts [20,50–55]. Importantly, the level of expression of these genes was significantly reduced in the peripheral blood cells and lungs after successful completion of anti-TB treatment (i.e., bacteriological cure) of the patients [20,50–55].

#### VDR signaling network

Of the SDEG, a subset of 65 genes was associated with the VDR signaling network in all the tested lesion types (S2 Fig and S5 Table). Expression of 49 genes was up regulated and 16 genes were down regulated in the AFB-rich lesions. Similar numbers of genes were expressed in both AFB-scarce and fibrotic lesions (48 genes up regulated and 17 genes down regulated). Interestingly, expression of *IL15*, a crucial mediator of VDR-signaling network, and *HTT*, *SPP1/OPN* and *CTNNB1*was up regulated only in the AFB-scarce lesion. IL-15 is crucial for linking the TLR-mediated innate signaling with the adaptive immune response and VDR-mediated antibacterial response during Mtb infection [47,56]. Expression of IL15 has also been shown to be induced upon activation of human primary monocytes with IFN-g or TLR2 treatment, which leads to VDR-mediated killing of Mtb [47]. Similar to IL-1, OPN has been shown to antimicrobial activities of human monocyte-derived macrophages through induction of reactive oxygen species [57]. In mice, OPN has been shown to regulate the recruitment and activation of macrophages during pulmonary granuloma formation and OPN-deficient mice were impaired for control of mycobacteria [58]. In addition, induction of OPN was observed during Mtb infection of human alveolar macrophages, and expression of OPN was reported in the lung sections of human patients with TB [59,60]. Moreover, an inverse correlation was observed in human TB patients between the level of OPN and the severity of disease and death due to disease [60]. Taken together, our data analyses suggest that differential expression of specific genes in the VDR signaling is associated with the characteristics of different lesions. For example, unique up regulation of *IL15* and *SPP1*/*OPN* can contribute to the reduced bacillary load observed in the AFB-scare, non-progressive lesion, compared to AFB-rich granuloma.

#### IL-17 interaction network

In the present study, we found the expression of a subset of 21 SDEG involved in IL-17 interaction network in AFB-rich, AFB-scarce and fibrotic lung lesions (S4 Fig and S5 Table). Majority of the SDEG in this network codes for cytokines and chemokines (*CCL2*, *CCR1*, *CXCL1*, *CXCL12*, *CXCL3*, *CXCL5*, *CXCL8*, *IL17RA*, *IL1A* and *IL1B*), and transcriptional regulators (*ATF2*, *BATF*, *CREBBP*, *HIF1A*, *IRF8*, *JUN* and *TRAF3*) that are actively involved in the regulation of IL-17 signaling in immune cells. Among these SDEG, expression of about 38%, including *IL17RA* were up regulated in all the tested lesion types. While expression of *BATF* and *CXCL12* were up regulated only in fibrotic granuloma, *MMP9* and *JUN* were up regulated in AFB-scare granuloma and *RBPJ* was up regulated exclusively in AFB-rich lesion. The IL-17 family of cytokines and their receptors, such as IL-17RA, are expressed by various host immune cells, including lymphocytes [61]. These molecules have also been shown to be important for the host immunity against infection and inflammation [61,62]. In NHP model of pulmonary TB, granulomas with less or no bacteria (sterile lesions) had higher levels of IL-17, compared to lesions with high bacterial load [63]. Similarly, IL-17RA knock-out mice showed defective liver fibrosis, had attenuated granulomatous inflammation and reduced expression of *CXCL1* during infection with *S*. *japonicum* [64]. These observations are consistent with our findings in human TB patients that showed elevated expression of *IL17RA*, *CXCL1* and *CXCL12* in the fibrotic nodular granulomas, compared to cavitary lesions in the lungs.

### Host gene expression in cavitary granulomas varies with bacillary content

Our histological analysis showed a difference in the bacillary load in the two cavitary lesions studied. Therefore, we examined whether the bacillary load can impact the level of immune activation within similar lesions. In this preliminary analysis with a clearly limited number of samples, the gene transcripts that were enriched and differentially regulated between the two cavitary granulomas were compared. The AFB-rich cavitary granuloma had about twice the number of SDEG than the AFB-scarce lesion (n = 7,261 versus 3,781). In addition, ~ 90% of the SDEG in the AFB-scarce lesion were also expressed in the AFB-rich cavitary granuloma and more than 60% of these genes were up-regulated in both granulomas. Thus, despite a similar immune cell distribution, a difference in the number and level of expression of SDEG was noted between AFB-rich and AFB-scarce cavitary granulomas.

To determine the effect of Mtb load on the molecular correlates of local immune regulation in lung cavitary granulomas, we performed networks/pathway analysis of SDEG from AFB-rich or AFB-scarce cavitary lesions. Our analysis showed that networks/pathways associated with lysosomal functions and cytokine responses to infection, including canonical interferon (IFN) signaling pathways were highly differentially regulated between the AFB-rich and AFB-scarce cavitary lesions (Table 3 and S6 Table).

#### Differential regulation of canonical IFN signaling pathway in AFB-rich or AFB-scarce cavitary granulomas

**Fig 4:**
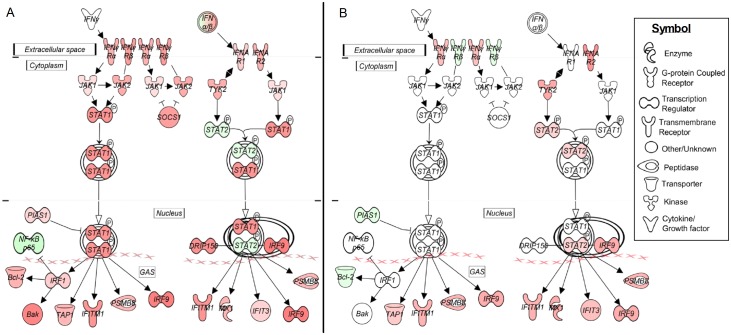
Differential regulation of interferon signaling pathway in the cavitary granulomas with different bacterial loads. Expression pattern and interaction of SDEG involved in canonical IFN signaling pathway in the cavitary granulomas with numerous (A) or scanty (B) AFB. For both (A) and (B), the up-regulated SDEG are in red and down-regulated SDEG are in green and the intensity of the color is proportional to their expression level (i.e., stronger expression is represented as dark shades).

Because of its biological significance to TB pathogenesis, we analyzed the expression pattern of SDEG in IFN pathway. Of the SDEG, a subset of 26 genes was associated with the IFN signaling pathway (Fig 4 and S6 Table). Expression of the majority of the IFN signaling genes (n = 23) was significantly up-regulated in the AFB-rich cavity (Fig 4A), compared to only 10 genes in the AFB-scanty cavity, while a similar number (n = 3) of genes was down-regulated in both lesions (Fig 4B). Importantly, several of the key genes in the IFN pathway, including *STAT1*, *SOCS1*, *JAK2*, *IRF1*, *MED14*, *IFNB1*, *PTPN6*, *IFNL3*, *RAF1*, *IFNAR1* and *JAK1* were significantly up-regulated only in the AFB-rich cavitary granuloma.

Taken together, our findings suggest an association between the maturation state of a lesion and the level of immune-stimulation. Thus, cavitary lesions with numerous AFB appeared to be immunologically more active, while the fibrotic nodules and other lesions with scanty or no AFB appeared less active. Consistent with this observation, a positive correlation between the antigenic load in the granulomas and the recruitment and, activation of leukocytes during TB pathogenesis has been reported previously [9,12,16,21,42,65,66]. Furthermore, in support of our findings, the elevated expression levels of disease-induced MMP-1, CCL-3,CXCL-8 and Type 1 IFN in the sputum and plasma of active TB patients has been reported to be significantly decreased to basal levels after 4–6 months of anti-TB drug treatment, which reduces the net bacillary load [19,26,67,68].

### Blood biomarkers of active TB are only partly represented in the lung granulomas

**Fig 5:**
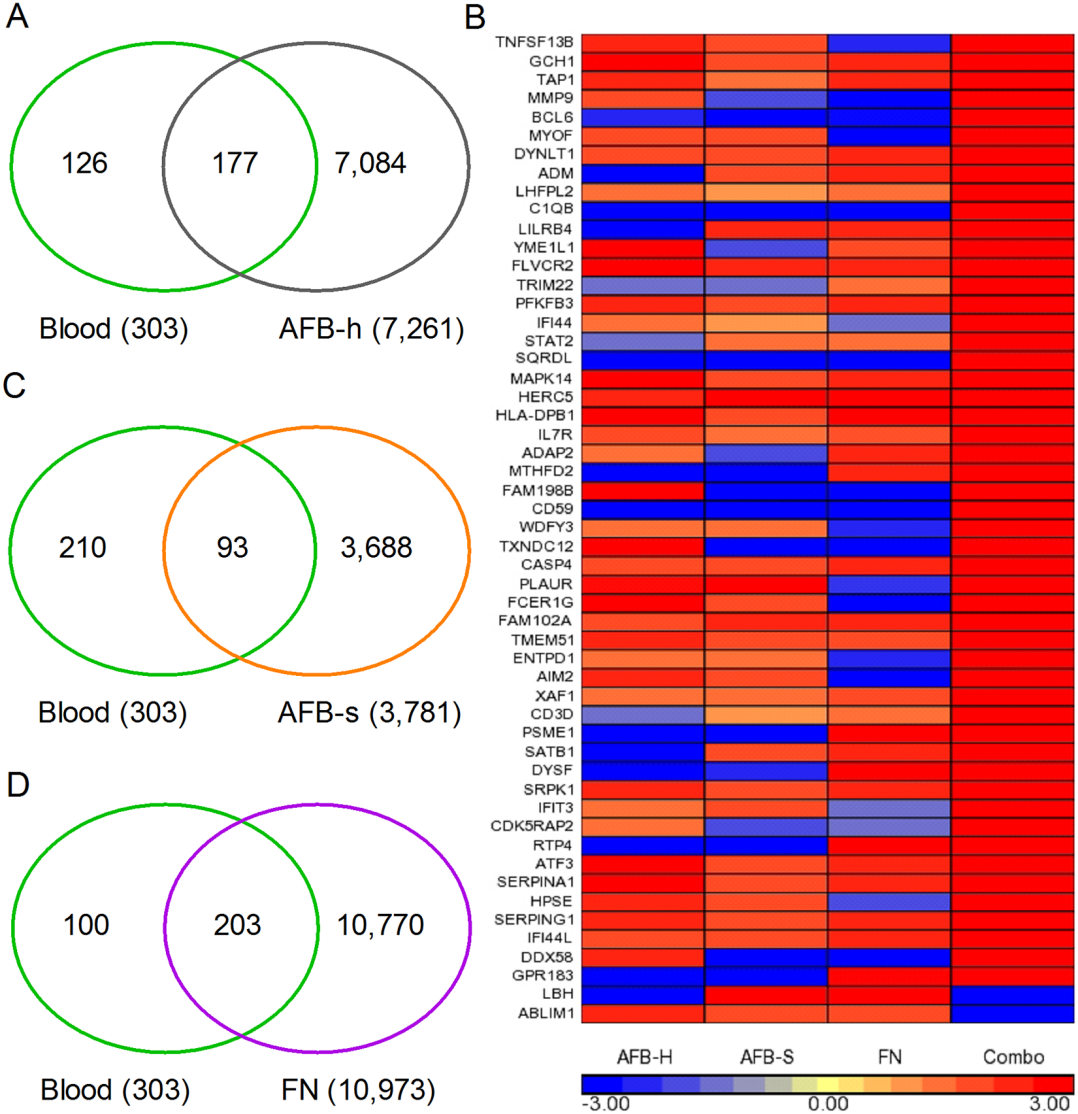
Expression of blood biomarkers of active TB in different TB granulomas. (A). Venn diagram showing comparison of blood TB biomarker profile (green circle) to SDEG from cavitary lung lesion with high AFB (AFB-h; grey circle). (B). Intensity plot of blood TB biomarker genes expressed in cavitary granulomas with scanty (AFB-s) or high (AFB-h) bacillary load, fibrotic nodule (FN) or common to all lesions versus pooled lung transcriptome (Combo). The up-regulated SDEG are in red and down-regulated SDEG are in blue and the intensity of the color is proportional to their expression level (i.e., stronger expression is represented as dark shades). Expression pattern in Combo is sorted in descending order (top to bottom). Scale bar ranges from +3 (red) to -3(blue). (C). Venn diagram showing comparison of blood TB biomarker profile (green circle) to SDEG from cavitary granuloma containing scarce bacillary load (AFB-s; orange circle) in the TB lungs. (D). Venn diagram showing comparison of blood TB biomarker profile (green circle) to SDEG from fibrotic nodule (FN; purple circle) in the TB lungs. For A, C and D, numbers in parenthesis indicate total number of SDEG.

Expression patterns of host genes identified in the blood have been used as biomarkers to differentiate active TB from LTBI [20,26,50–55,69–71]. Recently, Berry et.al, described a neutrophil-based host biosignature of TB, using the peripheral blood transcriptome of active TB patients, compared to latently-infected individuals [54]. We compared the expression profile of the blood biosignature from the Berry study to the lung granuloma transcriptome from our present study, to determine the concordance between the systemic and local host response to Mtb infection and/or disease. Of the 393 genes in the blood biosignature, 105 (~27%) were identified as significantly differentially expressed in the pooled TB lung granuloma (cavities + fibrotic nodules) transcriptome (S2 Fig and S7 Table). Of the 105 genes, 84 (~88%) were expressed in the same direction (up-regulated) in both the blood and lung granulomas of TB patients. To determine the contribution of the individual lung TB granulomas to the expression pattern found in blood, we compared the blood biomarker profile to the transcriptome of our three lesion types (Fig 5 and S7 Table). Of the 393 genes in the blood biomarker, 177 (~45%), 93 (~24%) and 203 (~52%) genes were similarly differentially regulated in the AFB-rich and AFB-scarce cavitary granulomas and, fibrotic lung lesions, respectively (Fig 5A, 5C and 5D). In these lesions, expression of 83 genes was shared with the blood TB biomarker profile (S7 Table). Of note, a different pattern of up-regulated genes was seen in each of the lung lesion studied. When the 83 common genes found in the blood biomarker and in the different lesions were compared to the gene expression pattern of the pooled lung transcriptome, 53 genes were similarly differentially regulated (Fig 5B). However, 37, 36 and 34 of these genes were up-regulated in the AFB-rich and AFB-scarce cavitary granulomas and, fibrotic lung lesions, respectively, while 51 genes were up-regulated in the pooled lung transcriptome data (Fig 5B and S7 Table).

Thus, a partial transcriptional pattern (~14%) from the pooled lung granulomas corresponded to the molecular signature identified in the blood of patients with active TB. However, the majority (> 80%) of genes expressed in the granulomas were unique to specific types of lesion and were either not represented or not significant in the blood profile of TB patients, which is consistent with previous reports [41,72,73]. Taken together, these observations suggest that the blood gene expression pattern represents transcriptional sampling of different types of granulomas present at any time in the TB lungs and is unlikely to adequately reflect disease. Our results are also supported by a more elaborate study in a NHP model of pulmonary TB, which showed both lesion-specific immune response as well as poor representation of lung immune response in the blood [63]. As shown in the present study, the micro-environment of individual granulomas, including distribution of lymphocytes, their activation status as well as the overall gene expression profiles, was significantly different from lesion to lesion [14]. Although the number of samples studied was very limited, the present observations suggest that the state of maturation/differentiation of each granuloma is likely to contribute to the pattern of immune activation seen in the different microenvironment. A more extensive analysis of a larger number of different types of granulomas will be important to delineate the specific local immune environments and their contribution to disease in pulmonary TB.

## Conclusions

In conclusion, we report the molecular correlates of the host immune response associated with the heterogeneity of granulomas in patients with chronic pulmonary TB. Our results suggest that the diversity of TB lung lesions is associated with differential immune activation in the various micro-environments, determined at least in part, by the local leukocyte response to the bacillary load. The localized immune transcriptional profiles associated with various types of TB granulomas described in this study should inform our understanding of the complex nature of host-pathogen interactions and may have utility for identifying surrogates of disease progression/control that track with bacterial burden. However, the varying microenvironment of TB granulomas, in concert with differential activation status of immune cells at the site of infection warrants transcriptional analysis of large numbers of human TB lung biopsies to define such biomarkers of disease progression.

## Supporting Information

S1 Fig H&E stained section of uninvolved lung tissue from active TB patient. Note the presence of small numbers of alveolar macrophages (inset) and few T cells.
(TIF)

S2 Fig Intensity plot of genes associated with VDR signaling network. Expression pattern is sorted in alphabetical order. Scale bar ranges from +5 (red) to -5 (blue).
(PDF)

S3 Fig Intensity plot of genes associated with IL-17 network. Expression pattern is sorted in alphabetical order. Scale bar ranges from +5 (red) to -5 (blue).
(PDF)

S4 Fig Intensity plot of blood TB biomarker genes expressed in pooled lung transcriptome. Expression pattern is sorted in descending order (top to bottom). Scale bar ranges from +5 (red) to -5 (blue).
(TIF)

S1 Table Canonical pathways activated in lung TB granulomas. List of canonical pathways affected by SDEG identified in this study.
(DOC)

S2 Table Top 15 most highly differentially expressed regulator genes in lung TB granulomas. The SDEG were selected based on their level of expression.
(DOC)

S3 Table List of SDEG common to fibrotic nodules and cavitary lesions. The total and differential SDEG between fibrotic and cavitary granulomas are shown in Fig 2.
(XLS)

S4 Table SDEG involved in immune cell movement, STAT1 mediated T cell activation and Fibrosis/wound healing networks. Interaction among member genes in this pathway is shown in Fig 3.
(XLS)

S5 Table SDEG involved in VDR signaling and IL17 networks. Gene expression pattern is sorted in alphabetical order.
(XLS)

S6 Table SDEG involved in canonical IFN pathway. Interaction among member genes in this pathway is shown in Fig 4.
(XLS)

S7 Table Comparison of blood biomarker of TB with lung granulomas. Summary of common and unique genes in various types of lung granulomas is shown in Fig 5.
(XLS)
