# Supplementary figures and images for: Lesion-Specific Immune Response in Granulomas of Patients with Pulmonary Tuberculosis: A Pilot Study
Source: PLoS One. 2015 Jul 2;10(7):e0132249. doi: 10.1371/journal.pone.0132249 (PMC4489805; doi:10.1371/journal.pone.0132249)

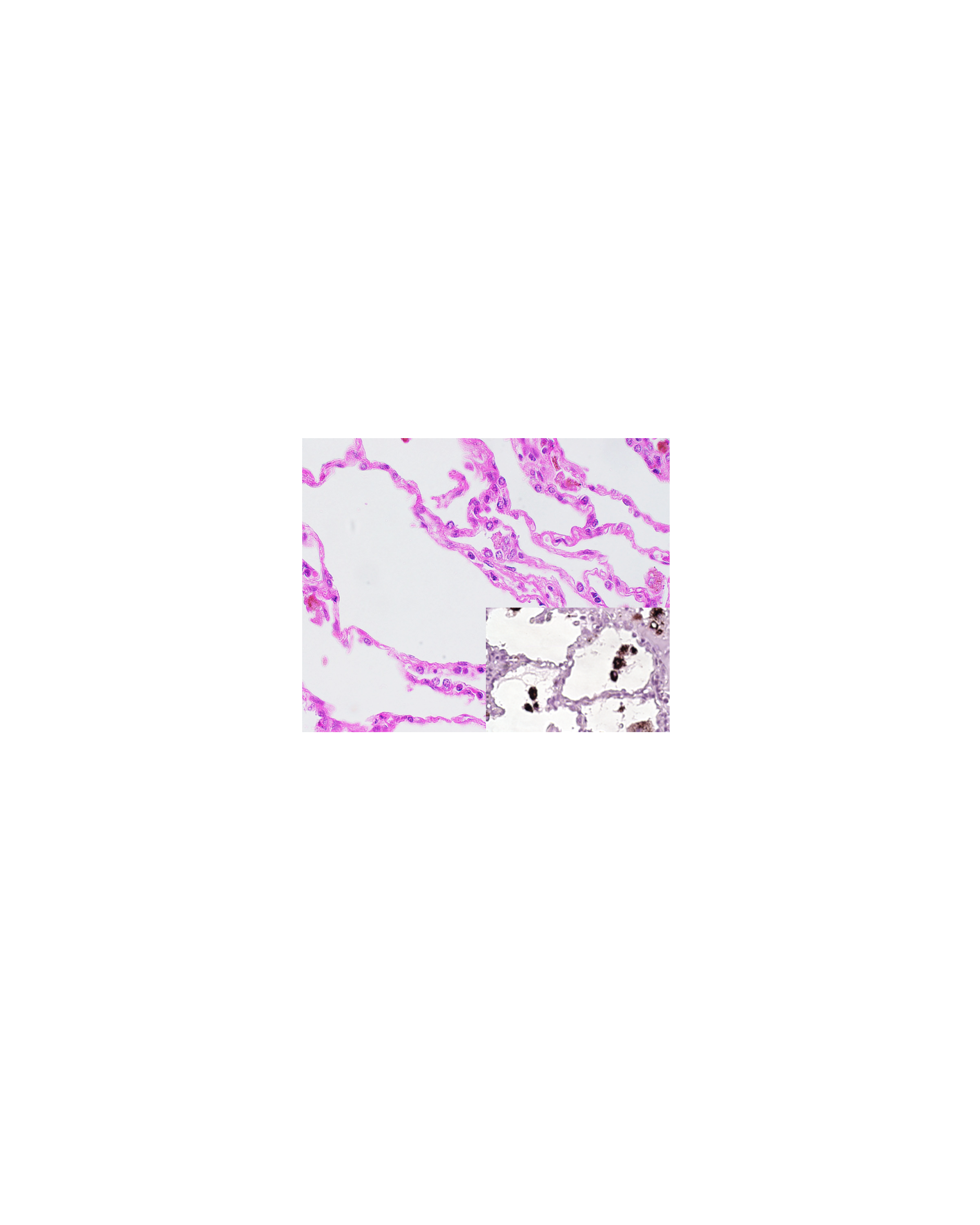

Supplement: S1 Fig — Note the presence of small numbers of alveolar macrophages (inset) and few T cells. (TIF) [file pone.0132249.s001.tif]

## VDR signaling

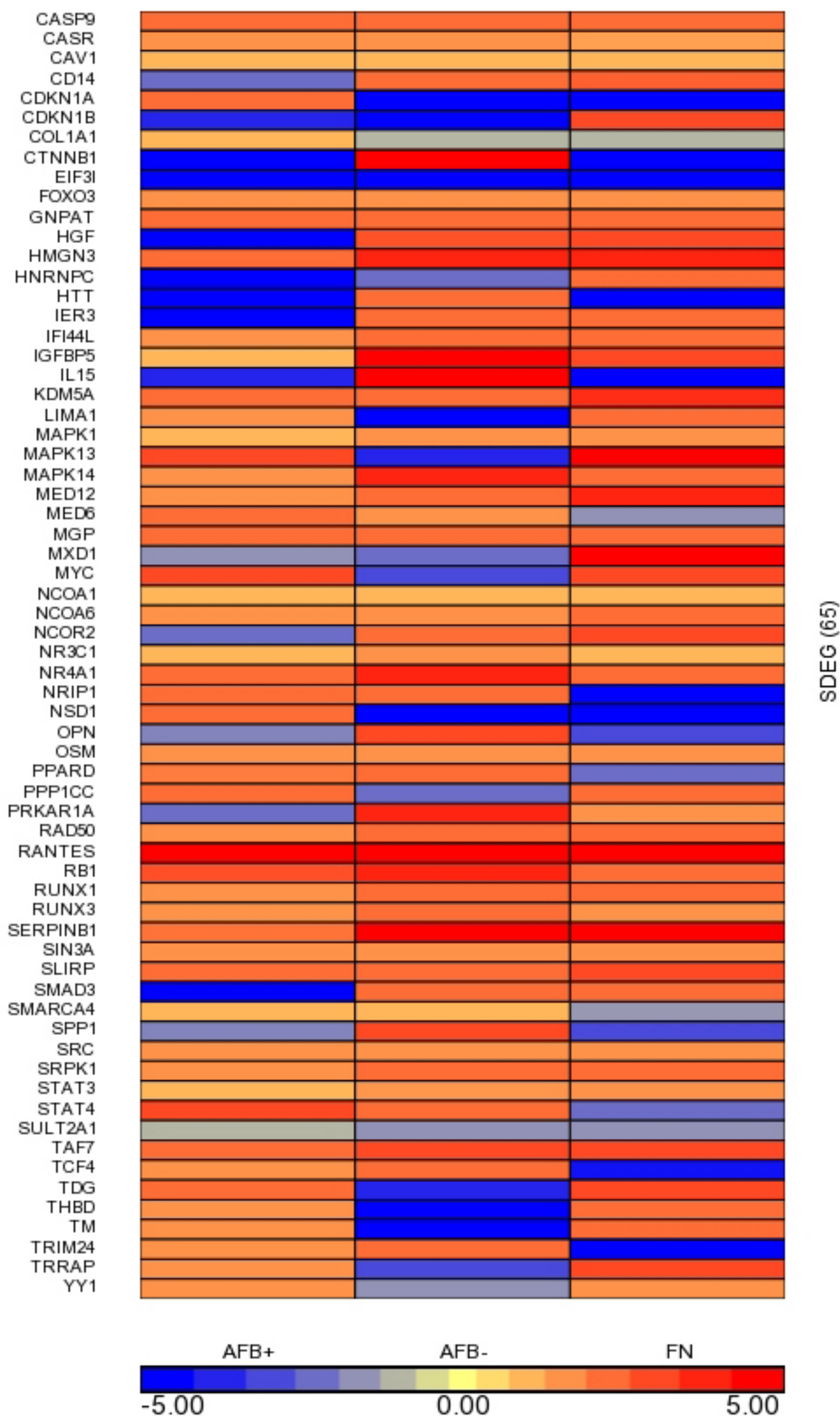

Supplement: S2 Fig — Expression pattern is sorted in alphabetical order. Scale bar ranges from +5 (red) to -5 (blue). (PDF) [file pone.0132249.s002.pdf]

# IL-17 interaction network

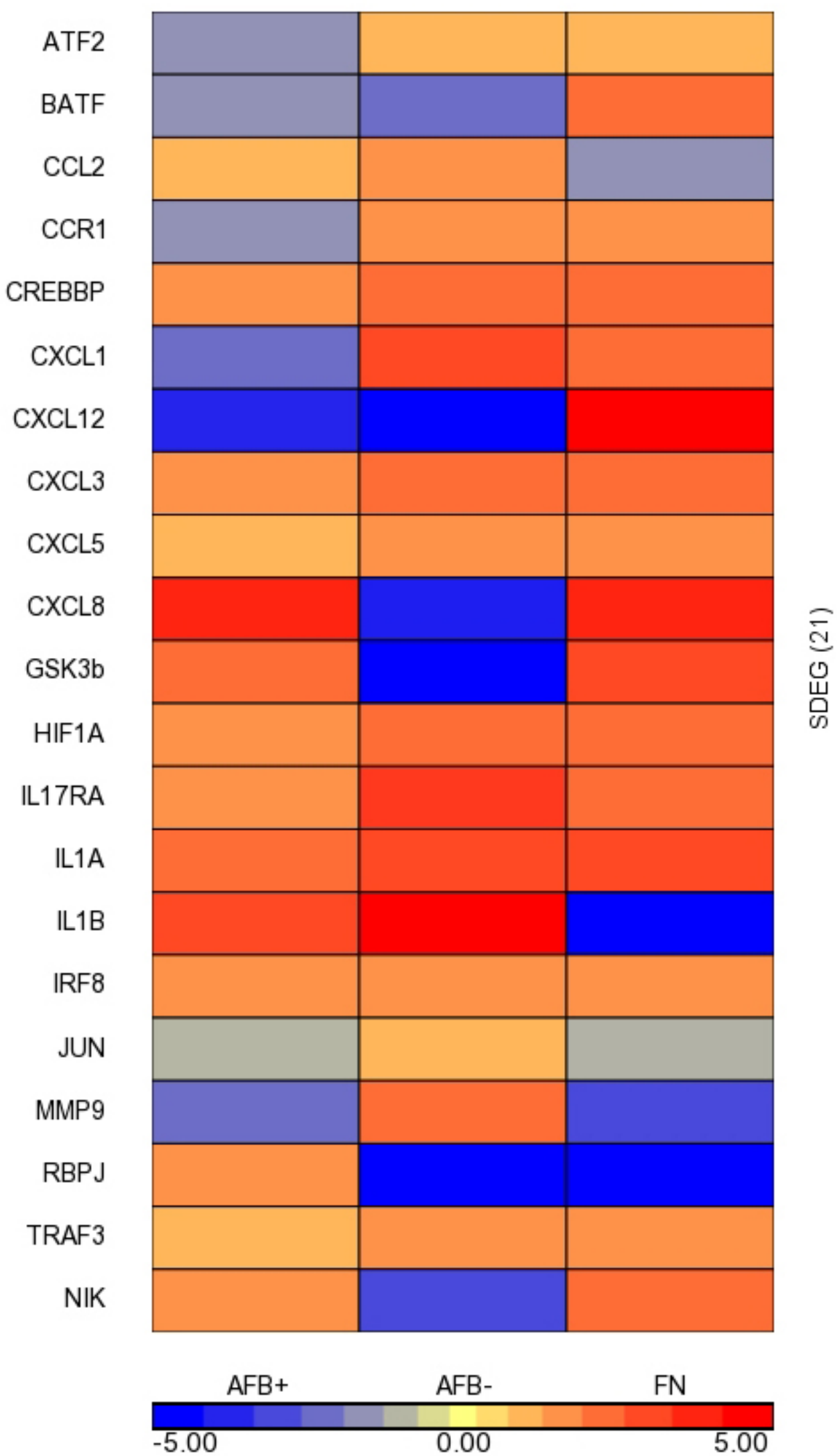

Supplement: S3 Fig — Expression pattern is sorted in alphabetical order. Scale bar ranges from +5 (red) to -5 (blue). (PDF) [file pone.0132249.s003.pdf]

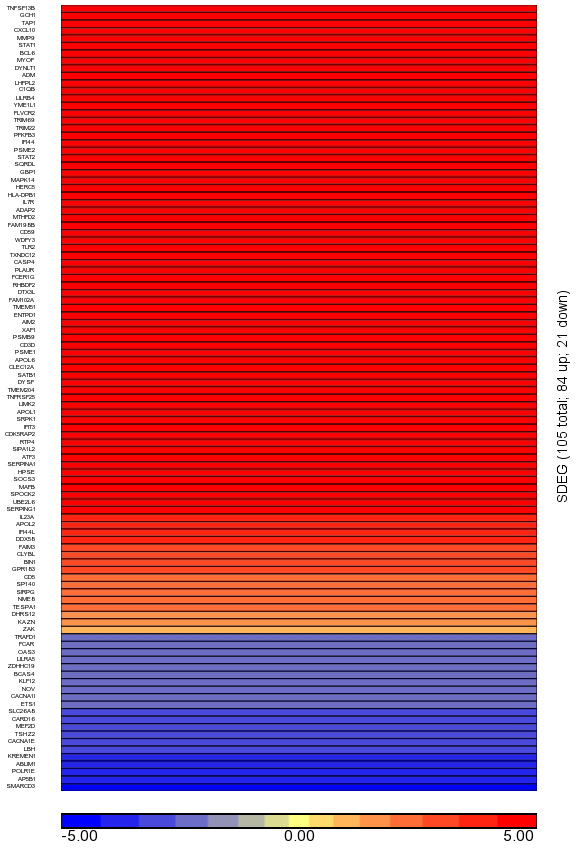

Supplement: S4 Fig — Expression pattern is sorted in descending order (top to bottom). Scale bar ranges from +5 (red) to -5 (blue). (TIF) [file pone.0132249.s004.tif]
