# Supplementary material for: Lesion-Specific Immune Response in Granulomas of Patients with Pulmonary Tuberculosis: A Pilot Study
Source: PLoS One. 2015 Jul 2;10(7):e0132249. doi: 10.1371/journal.pone.0132249 (PMC4489805; doi:10.1371/journal.pone.0132249)
Supplement: S1 Table — List of canonical pathways affected by SDEG identified in this study. (DOC) [file pone.0132249.s005.doc]

Supplementary Table 1. Canonical pathways activated in lung TB granulomas

| **No.** | **Pathways** | **-log(p-value)** |
| --- | --- | --- |
| 1 | Molecular Mechanisms of Cancer | 1.22E+01 |
| 2 | B Cell Receptor Signaling | 1.02E+01 |
| 3 | Production of Nitric Oxide and Reactive Oxygen Species in Macrophages | 8.99E+00 |
| 4 | PKCθ Signaling in T Lymphocytes | 8.47E+00 |
| 5 | Integrin Signaling | 8.40E+00 |
| 6 | Protein Ubiquitination Pathway | 8.04E+00 |
| 7 | HER-2 Signaling in Breast Cancer | 7.65E+00 |
| 8 | Role of NFAT in Regulation of the Immune Response | 7.45E+00 |
| 9 | CD28 Signaling in T Helper Cells | 7.38E+00 |
| 10 | Antigen Presentation Pathway | 7.14E+00 |
| 11 | IL-8 Signaling | 6.95E+00 |
| 12 | Gαq Signaling | 6.88E+00 |
| 13 | Hepatic Fibrosis / Hepatic Stellate Cell Activation | 6.72E+00 |
| 14 | PI3K Signaling in B Lymphocytes | 6.72E+00 |
| 15 | CXCR4 Signaling | 6.61E+00 |
| 16 | Natural Killer Cell Signaling | 6.43E+00 |
| 17 | Leukocyte Extravasation Signaling | 6.39E+00 |
| 18 | Cardiac Hypertrophy Signaling | 6.37E+00 |
| 19 | iCOS-iCOSL Signaling in T Helper Cells | 6.31E+00 |
| 20 | Fcγ Receptor-mediated Phagocytosis in Macrophages and Monocytes | 6.30E+00 |
