# Supplementary material for: Lesion-Specific Immune Response in Granulomas of Patients with Pulmonary Tuberculosis: A Pilot Study
Source: PLoS One. 2015 Jul 2;10(7):e0132249. doi: 10.1371/journal.pone.0132249 (PMC4489805; doi:10.1371/journal.pone.0132249)
Supplement: S2 Table — The SDEG were selected based on their level of expression. (DOC) [file pone.0132249.s006.doc]

**Supplementary Table 2.** Top 15 most highly differentially expressed regulator genes in lung TB granulomas

| **Regulator** | | **Fold Change** | **Molecule Type** | **z-score** | **p-value** |
| --- | --- | --- | --- | --- | --- |
| ***Up regulated*** | |  |  |  |  |
| *CXCR4* | | 173.721 | G-protein coupled receptor | 0.911 | 8.02E-03 |
| *CCL3* | | 148.839 | cytokine | 1.367 | 6.24E-03 |
| *TNFSF13B* | | 116.506 | cytokine | 1.558 | 3.75E-04 |
| *CXCL8* | | 110.317 | cytokine | 3.363 | 5.17E-03 |
| *MAP3K5* | | 87.909 | kinase | 2.219 | 8.03E-02 |
| *MMP1* | | 84.694 | peptidase | 2.383 | 1.51E-01 |
| *CHI3L1* | | 84.125 | enzyme |  | 1.81E-04 |
| *CTGF* | | 83.503 | growth factor | 1.885 | 2.56E-03 |
| *PSEN2* | | 62.286 | peptidase | -0.532 | 1.72E-03 |
| *MMP9* | | 61.719 | peptidase | 0.206 | 3.43E-02 |
| *DAB2* | | 61.65 | other | -1.176 | 3.00E-02 |
| *CYP1B1* | | 58.951 | enzyme | 0.684 | 1.91E-03 |
| *STAT1* | | 58.288 | transcription regulator | 4.753 | 2.13E-04 |
| *BCL6* | | 56.427 | transcription regulator | -1.038 | 8.14E-04 |
| *SGK1* | | 54.487 | kinase | -1.189 | 8.02E-03 |
| ***Down regulated*** | | |  |  |  |
| *FOXC1* | -9.419 | | transcription regulator | -1.522 | 1.31E-02 |
| *PSMD4* | -8.875 | | other | 0 | 1.23E-02 |
| *FABP4* | -7.913 | | transporter | -0.103 | 3.86E-02 |
| *AGER* | -7.047 | | transmembrane receptor | 1.894 | 6.51E-02 |
| *PIN1* | -6.905 | | enzyme | 0.757 | 2.51E-02 |
| *AKT3* | -6.418 | | kinase | -0.707 | 3.86E-02 |
| *NFIX* | -5.415 | | transcription regulator | 0.194 | 5.96E-03 |
| *HGF* | -5.333 | | growth factor | 2.95 | 1.59E-09 |
| *ERG* | -4.955 | | transcription regulator | 2.989 | 3.66E-12 |
| *ERBB3* | -4.646 | | kinase | 0.805 | 8.91E-05 |
| *EGFR* | -4.37 | | kinase | 3.21 | 4.30E-08 |
| *IFNA21* | -4.213 | | cytokine | 1.951 | 4.23E-01 |
| *ATN1* | -4.189 | | transcription regulator | 0 | 1.81E-03 |
| *NEUROG1* | -4.112 | | transcription regulator | -1.633 | 1.00E-02 |
| *ITGB3* | -4.072 | | transmembrane receptor | 1.313 | 6.72E-05 |
